# Two near‐complete assemblies reveal R‐subgenome structural and centromeric divergence associated with reproductive isolation in hexaploid triticale

**DOI:** 10.1002/imt2.70155

**Published:** 2026-07-13

**Authors:** Yang Liu, Wanna He, Chunhui Wang, Congyang Yi, Tianxin Guo, Congle Zhu, Qian Liu, Fangpu Han

**Affiliations:** ^1^ State Key Laboratory of Seed Innovation, Institute of Genetics and Developmental Biology Chinese Academy of Sciences Beijing China; ^2^ College of Advanced Agricultural Sciences University of Chinese Academy of Sciences Beijing China

## Abstract

We present two near‐complete triticale genome assemblies and perform pan‐centromere analyses across >200 haplotypes from parental species, synthetic allopolyploids, cultivars, and 337 resequenced accessions, revealing a shared chromatin framework underlying functional centromeres in all three subgenomes, where wheat‐ and rye‐derived CENH3 co‐occupy divergent centromeric repeats. Postpolyploid evolution drives extensive centromere remodeling, including size convergence, repeat turnover, and locus repositioning, with the R subgenome emerging as the primary source of structural and centromeric divergence. In F_1_ hybrids, R‐subgenome structural and centromeric divergence is associated with meiotic abnormalities, including chromosome misalignment, lagging chromosomes, and micronuclei formation, suggesting a link between centromere variation and reduced meiotic stability.

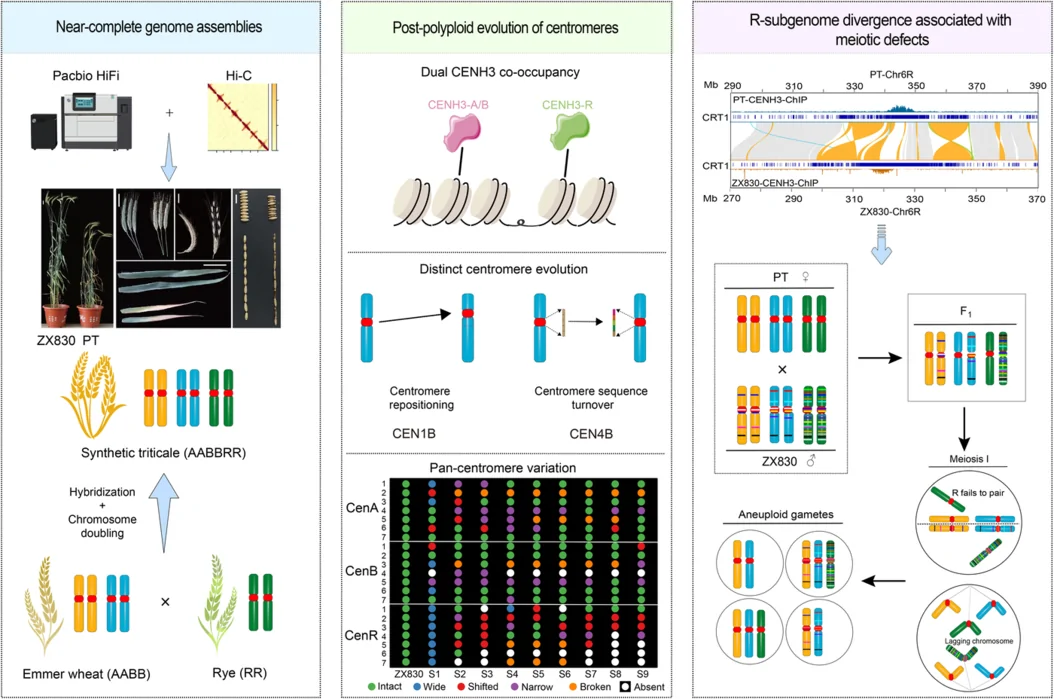

To the Editor,

Allopolyploidization is a major evolutionary force underlying the origin of many important crops, including wheat, cotton, canola, and oat. By merging divergent genomes into a single nucleus, allopolyploid formation creates immediate challenges for chromosome pairing, segregation, and long‐term genome stabilization [1, 2]. Among the genomic regions most directly linked to these processes are centromeres, which ensure accurate chromosome inheritance through kinetochore assembly and spindle attachment [3, 4]. Despite their essential role, centromeres are among the fastest‐evolving regions of eukaryotic genomes and are typically composed of highly repetitive DNA, including satellite repeats and centromere‐associated retrotransposons [5, 6, 7, 8, 9, 10]. How centromeres from divergent parental species become functionally compatible and subsequently diversify following allopolyploidization remains poorly understood.

Hexaploid triticale (**×** *Triticosecale* Wittmack, AABBRR), generated through hybridization between tetraploid wheat (*Triticum turgidum*, AABB) and rye (*Secale cereale*, RR) [11, 12, 13], provides an ideal model for addressing these questions. As the first successfully commercialized synthetic cereal crop, triticale initially exhibited severe meiotic instability, reduced fertility, and extensive chromosomal abnormalities [14]. Although modern breeding has substantially improved agronomic performance, residual genome instability and structural variation remain widespread among triticale accessions, suggesting that genome stabilization is still ongoing [15]. This dynamic genomic landscape positions triticale as a powerful model for investigating centromere behavior, adaptation, and diversification across both the establishment and long‐term evolution of an allopolyploid genome. Previous cytological studies documented extensive karyotypic diversity in triticale, including aneuploidy, chromosome substitutions, and large chromosomal rearrangements [15]. However, the absence of species‐specific reference genomes has limited detailed investigation of repeat‐rich centromeric and pericentromeric regions. Consequently, the organization, evolution, and population‐level diversification of functional centromeres in triticale have remained largely unexplored. Here, we generated near‐complete genome assemblies for two divergent hexaploid triticale lines and integrated them with functional centromere mapping, comparative genomics, pan‐centromere analysis, and population genomics to investigate centromere evolution and reproductive isolation in an allopolyploid genome.

## NEAR‐COMPLETE GENOME ASSEMBLY AND GENE ANNOTATION OF THE HEXAPLOID TRITICALE CULTIVAR ZX830

**Figure 1:**
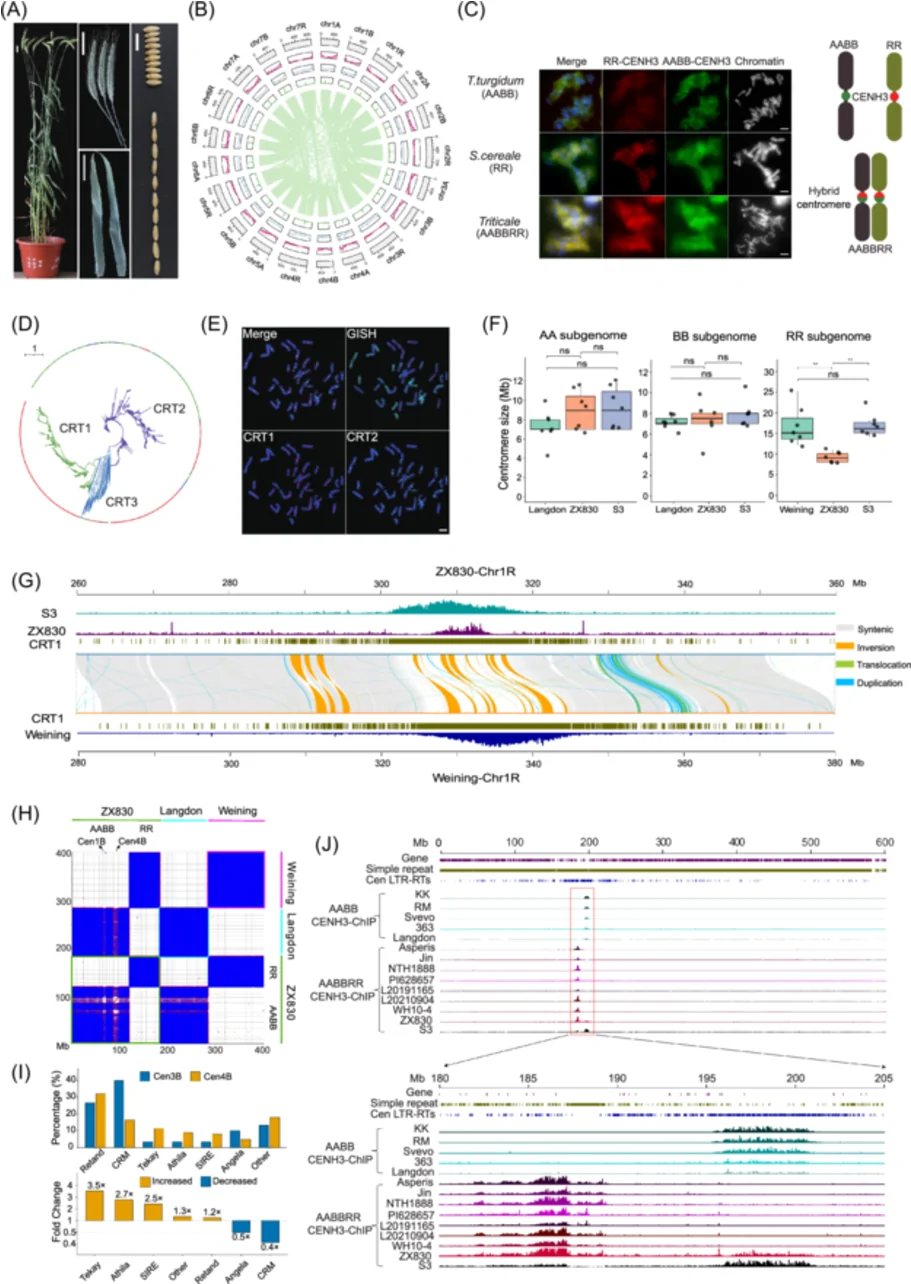
Near‐complete genome assembly and dynamic centromere evolution in hexaploid triticale. (A) Representative plants of the hexaploid triticale line ZX830 grown under field conditions. Shown are whole plants, spike and leaf morphology at the heading stage, and grain length and width of mature seeds. Scale bars, 5 cm (whole plants, spikes, and leaves) and 1 cm (mature seeds). (B) Circos representation of the assembled genome. Tracks from the outer to the inner circles display chromosomes, gene density, LTR density, CENH3 ChIP‐seq signal intensity, and syntenic blocks. (C) Immunofluorescence detection of subgenome‐specific CENH3 in parental species and hexaploid triticale. Wheat‐specific (AABB‐CENH3) and rye‐specific (RR‐CENH3) antibodies label centromeres in *Triticum turgidum* (AABB) and *Secale cereale* (RR), respectively. In hexaploid triticale (AABBRR), both antibodies co‐localize at every centromere, demonstrating dual loading of wheat and rye CENH3. A schematic illustration summarizes centromere composition in parental genomes and triticale. Scale bars: 10 μm. (D) Phylogenetic reconstruction of centromere‐associated retrotransposons in triticale based on RT domain sequences from a randomly sampled subset of CRM elements. Three well‐supported centromeric retrotransposon lineages of triticale, CRT1–CRT3, are resolved. Colors in the outer ring denote subgenome assignment (R, red; A, green; B, light blue). (E) FISH using lineage‐specific LTR probes showing distinct centromeric localization patterns of CRT1 and CRT2. GISH signals are shown for subgenome identification. Scale bars: 10 μm. (F) Box plots summarizing centromere size distributions in the A, B, and R subgenomes of Langdon, Weining, ZX830, and S3. For each group, *n* = 7 centromeres corresponding to the seven chromosomes of the AA, BB, or RR subgenome. Statistical significance was assessed within each subgenome using one‐way ANOVA followed by post hoc pairwise comparisons with Benjamini‐Hochberg false discovery rate (FDR) correction for multiple testing; ns, not significant; *FDR‐adjusted *p* < 0.01. (G) Representative synteny analysis of the R subgenome between ZX830 and its rye progenitor Weining across an extended centromeric and pericentromeric region (100 Mb) on chromosome 1 R. Tracks from top to bottom show CENH3 ChIP‐seq signal in S3, CENH3 ChIP‐seq signal in ZX830, distribution of CRT1 elements in ZX830, pairwise syntenic relationships between ZX830 and Weining, distribution of CRT1 elements in Weining, and CENH3 ChIP‐seq signal in Weining. Colored ribbons indicate syntenic blocks, inversions, translocations, and duplications, as indicated. (H) Dot‐plot comparisons of centromeric regions between ZX830 and its parental genomes, Langdon and Weining, highlighting overall conservation of most centromeres and pronounced divergence at CEN1B and CEN4B. (I) Top: Relative abundance of major LTR retrotransposon lineages within the centromeric regions of the canonical centromere CEN3B and the divergent centromere CEN4B, as determined by lineage‐level classification of de novo‐identified repeat families. Bottom: Fold change in the abundance of major LTR retrotransposon lineages in CEN4B relative to CEN3B, highlighting lineage‐specific expansions and depletions. (J) Genome‐browser views showing CENH3 ChIP‐seq profiles across chromosome 1B in tetraploid AABB cultivars, hexaploid triticale lines, and a newly synthesized hexaploid triticale (S3), all mapped to the ZX830 reference genome. Tracks display gene annotation, simple repeats, centromere‐associated LTR retrotransposons, and CENH3 ChIP‐seq signals from individual accessions. The red box highlights the region encompassing the repositioned centromere (CEN1B). The lower panel shows a magnified view of the indicated interval. ANOVA, analysis of variance; CENH3, centromere‐specific histone H3; CRT1–CRT3, centromeric retrotransposon of triticale lineages 1–3; GISH, genomic in situ hybridization; LTR, long terminal repeat; RT, reverse transcriptase.

We first generated a near‐complete chromosome‐scale assembly for the representative triticale cultivar ZX830 using PacBio HiFi and Hi‐C sequencing. The final assembly spanned 15.9 Gb, with 99.7% of sequences anchored into 21 pseudochromosomes and a scaffold N50 of 755.7 Mb (Figure 1A,B and Figure S1A, Tables S1–S3). BUSCO, LAI, and Hi‐C interaction heatmap analyses demonstrated high completeness, continuity, and chromosome‐scale assembly accuracy, supporting the reliability of the assembly for centromere analysis (Figure S1B and Tables S4 and S5). Comparative synteny analyses further revealed strong collinearity between the A/B subgenomes of ZX830 and tetraploid wheat [16], as well as between the R subgenome and cultivated rye [17] (Figure S1C). Genome annotation showed that repetitive sequences accounted for more than 94% of the genome, with the R subgenome exhibiting particularly high transposable element content relative to the A and B subgenomes (Figure S2A,B). In contrast, gene density was substantially lower in the R subgenome, reflecting extensive expansion of repetitive intergenic regions (Figure S2C–E).

## CENTROMERES IN HEXAPLOID TRITICALE ARE DEFINED BY DUAL LOADING OF WHEAT AND RYE CENH3 ON EVOLUTIONARILY DIVERGENT PARENTAL SEQUENCES

To define functional centromeres in triticale, we developed subgenome‐specific antibodies against wheat‐ and rye‐derived CENH3 proteins (Figure S3A). Immunofluorescence and CENH3 ChIP‐seq analyses showed that both parental CENH3 variants co‐localized at all centromeres in triticale (Figure 1C and Figure S3B). We then performed mixed‐antibody CENH3 ChIP‐seq to define functional centromere boundaries across all 21 chromosomes (Figure S3C). Functional centromeres ranged from 6.9 to 12.1 Mb and were highly enriched in repetitive elements, particularly centromere‐associated LTR retrotransposons (Figure S4A and Table S6).

To further characterize centromeric repeat composition, we identified 118,130 full‐length LTR retrotransposons across the ZX830 genome (Figure S4A and Table S6). Classification of reverse transcriptase domains grouped these elements into seven major lineages that displayed pronounced subgenome‐biased accumulation and distinct chromosomal distributions. For example, Athila, SIRE, and Tekay elements were strongly enriched in the R subgenome, whereas CRM elements were predominantly concentrated in centromeric regions, while Ale elements were mainly distributed along chromosome arms (Figure S4B,C and Table S7). Comparative analyses revealed pronounced differences in centromeric repeat composition between the A/B and R subgenomes (Figure S5A). Phylogenetic reconstruction of centromere‐associated retrotransposons identified three major centromeric retrotransposon lineages in triticale, designated centromere retrotransposons of triticale (CRT1–CRT3). CRT1 was strongly enriched in the R subgenome and clustered closely with the rye‐specific Bilby family, whereas CRT2 predominated in the A and B subgenomes and was associated with wheat centromeric retrotransposons (Figure 1D and Figure S5B). Fluorescence in situ hybridization (FISH) further confirmed distinct subgenome‐specific distribution patterns of these repeats (Figure 1E). Thus, despite substantial divergence in underlying centromeric DNA composition, all triticale centromeres remain co‐occupied by both wheat and rye CENH3 proteins, indicating that epigenetic compatibility rather than repeat sequence identity is the major determinant of centromere function in triticale.

## CENTROMERE SIZE CONVERGENCE EMERGES DURING POSTPOLYPLOID EVOLUTION IN ZX830

To investigate how centromeres evolved following allopolyploidization, we compared triticale centromeres with those of tetraploid wheat and rye. Whereas centromeres from the A and B subgenomes remained largely comparable in size to those of tetraploid wheat, R‐subgenome centromeres were substantially contracted relative to rye centromeres (Figure 1F). To determine whether this contraction arose immediately after allopolyploidization or during subsequent evolution, we generated newly synthesized hexaploid triticale (S3 generation) and profiled CENH3 occupancy. Interestingly, the S3 synthetic triticale retained large rye‐like centromeres (Figure 1F,G), suggesting that major centromere contraction in the R subgenome was not established immediately after hybridization but instead emerged during subsequent postpolyploid evolution. However, because the S3 generation represents only an early stage following allopolyploid formation, we cannot exclude the possibility that additional centromeric remodeling occurred during later generations. Comparative synteny analyses further showed that these changes occurred without extensive chromosome‐scale disruption (Figure 1G and Figures S6 and S7), suggesting that the observed centromere size differences are primarily associated with altered CENH3 occupancy domains rather than large‐scale sequence loss. We note, however, that changes in CENH3 occupancy may also reflect chromatin remodeling or epigenetic regulation, and therefore do not necessarily indicate direct alterations in centromere functional strength.

## DISTINCT CENTROMERE EVOLUTIONARY TRAJECTORIES AT CEN4B AND CEN1B IN HEXAPLOID TRITICALE

Although most centromeres retained strong similarity to their parental counterparts, two B‐subgenome centromeres, CEN1B and CEN4B, displayed exceptional evolutionary trajectories (Figure 1H). Comparative analyses revealed pronounced divergence at both loci relative to tetraploid wheat centromeres. In CEN4B, canonical CRM‐type centromeric retrotransposons were strongly depleted and replaced by noncanonical *Gypsy* retrotransposon lineages, including *Retand* and *Tekay* elements, indicating extensive turnover of the centromeric repeat landscape (Figure 1I and Table S8). This atypical composition suggests relaxation of the selective constraints that normally maintain canonical centromeric repeats and highlights the remarkable plasticity of centromeric sequence organization in allopolyploid genomes.

CEN1B exhibited a different evolutionary pattern consistent with a centromere repositioning event (Figure 1J). In ZX830, the dominant CENH3‐enriched domain localized to a region distinct from the ancestral centromere found in tetraploid wheat. Residual CENH3 occupancy remained detectable at the ancestral locus, suggesting partial inactivation during establishment of the new centromere. Importantly, newly synthesized triticale retained CENH3 occupancy exclusively at the ancestral locus, indicating that centromere repositioning arose during postpolyploid evolution rather than immediately after allopolyploid formation (Figure 1J). Sequence analyses further revealed substantial expansion of trinucleotide repeats surrounding the newly established centromere region in ZX830, suggesting that local repeat amplification may have facilitated neocentromere formation (Figure S8A,B). Notably, this repositioning occurred without major chromosomal rearrangements (Figure S8C), highlighting the epigenetic plasticity of centromere identity during allopolyploid evolution.

## POPULATION STRUCTURE AND PAN‐CENTROMERE VARIATION REVEAL PRONOUNCED DIVERGENCE OF THE R SUBGENOME IN HEXAPLOID TRITICALE

**Figure 2:**
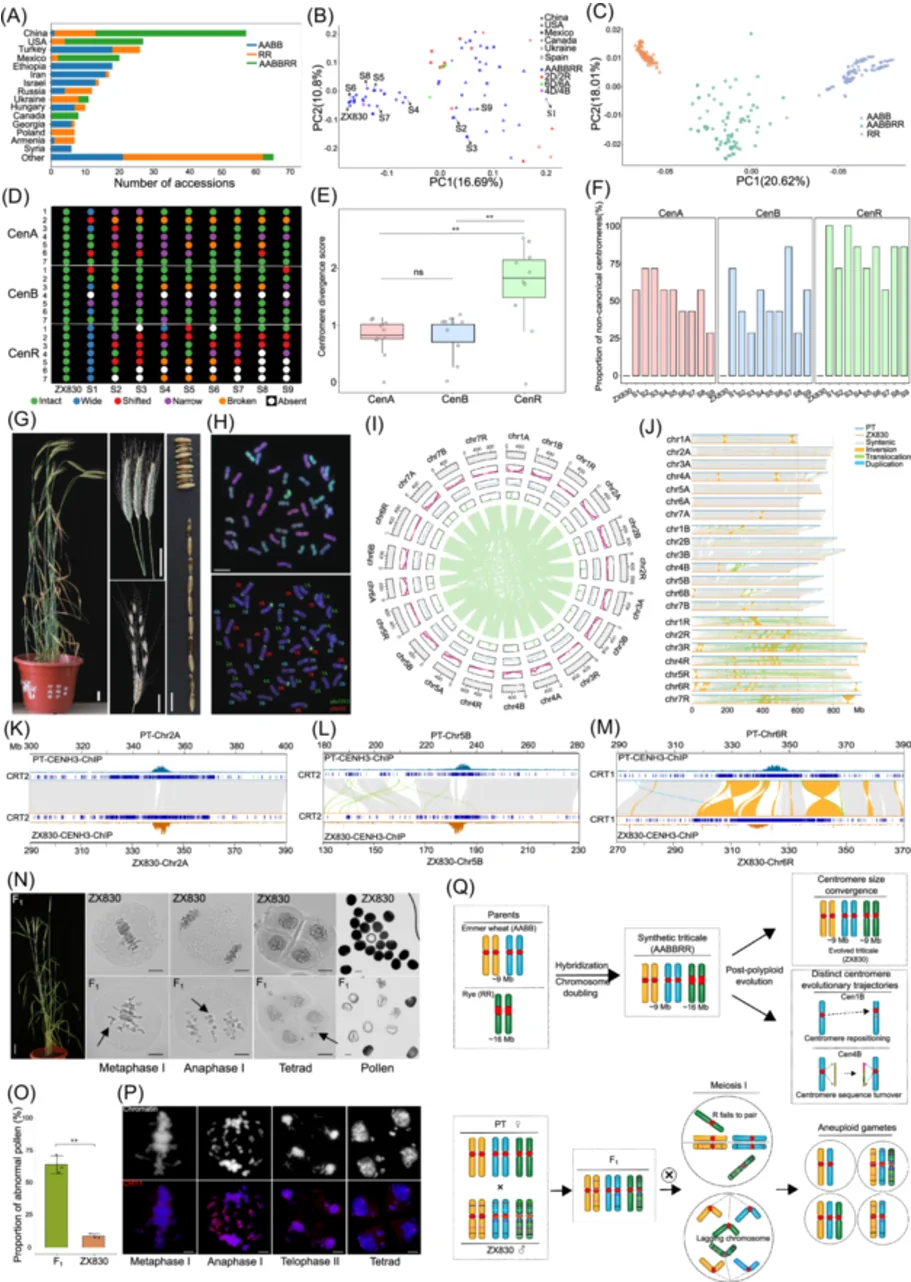
Population divergence, pan‐centromere variation, and R‐subgenome‐associated meiotic instability in hexaploid triticale. (A) Geographic distribution of the accessions analyzed in this study, including 99 triticale accessions, 122 tetraploid wheat accessions, and 116 rye accessions. (B) PCA of 81 stable hexaploid triticale accessions (63 canonical lines and 18 substitution lines) based on ~61.0 million high‐quality SNPs. The 10 accessions selected for pan‐centromere analysis (S1–S9 and ZX830) are highlighted in the PCA plot and correspond to the materials analyzed in panel (D). (C) PCA including hexaploid triticale and its parental species, tetraploid wheat and rye, based on genome‐wide SNPs uniformly mapped to the ZX830 reference genome. PC1 separates the three populations, whereas PC2 highlights asymmetric divergence of hexaploid triticale toward the wheat parent. (D) Summary matrix of centromere structural variation across 189 centromeres, categorized by CENH3 peak status, including intact, widened, narrowed, shifted, broken, or absent centromeres. Accessions are labeled S1–S9, where S1 corresponds to PT, S2 to PI628657, S3 to L20191165, S4 to L20210904, S5 to WH10‐4, S6 to Asperis, S7 to NTH1888, S8 to Jin, and S9 to Y70; accession details are provided in Supporting Information S1: Table S9. (E) Quantification of centromere divergence scores in the A, B, and R subgenomes based on the CENH3 peak states shown in panel (D). Centromere divergence scores were assigned as follows: intact = 0; widened or narrowed = 1; shifted = 2; broken = 3; and absent = 4. For each accession, the divergence score of each subgenome was calculated as the mean score of seven chromosome‐level centromeres. Each point therefore represents the accession‐level mean divergence score for one subgenome, rather than an individual centromere or technical replicate. ZX830 was used as the reference and was not included in this statistical comparison. For each subgenome, *n* = 9 accessions. Statistical significance was assessed using one‐way ANOVA followed by post hoc pairwise comparisons with Benjamini‐Hochberg FDR correction for multiple testing; ns, not significant; **FDR‐adjusted *p* < 0.01. (F) Proportion of noncanonical centromeres in the A, B, and R subgenomes across ten hexaploid triticale accessions. For each accession × subgenome combination, the proportion was calculated as *k*/7, where *k* represents the number of centromeres classified as noncanonical among the seven chromosome‐level centromeres in that subgenome. Noncanonical centromeres include widened, narrowed, shifted, broken, and absent CENH3‐enriched domains, as defined in panel (D). Because each value is based on a discrete binomial count of 0–7 centromeres, the bars are shown as accession‐level descriptive proportions rather than continuous measurements. (G) The phenotypes of the hexaploid triticale accessions PT, showing overall plant architecture, spikes, and seeds. Scale bars, 5 cm (whole plants and spikes) and 1 cm (seeds). (H) Karyotype analysis of PT confirming the expected hexaploid chromosome complement (AABBRR). Scale bars: 10 μm. (I) Genome‐wide overview of the PT assembly, showing 21 pseudochromosomes. Tracks from the outer to the inner circles display chromosomes, gene density, LTR density, CENH3 ChIP‐seq signal intensity, and syntenic blocks. (J) Whole‐genome synteny comparison between ZX830 and PT. While the A and B subgenomes display largely conserved collinearity, the R subgenome exhibits extensive structural divergence, including disrupted syntenic relationships and rearrangements. (K–M) Enlarged views of representative centromeric and pericentromeric regions comparing a 100‐Mb interval centered on the functional centromere between ZX830 and PT. The corresponding comparisons for all remaining chromosomes are shown in Supporting Information S1: Figure S15. (N) The phenotype of F_1_ hybrid. Scale bar: 15 cm. Comparison of meiotic chromosome behavior and pollen viability between ZX830 and F_1_ hybrid. Representative images show Metaphase I, Anaphase I, tetrads, and pollen grains. Arrows indicate chromosome bridges, lagging chromosomes and micronuclei in F_1_. Pollen viability was assessed by staining. Scale bars: 10 μm. (O) Proportion of abnormal pollen in F_1_ hybrids and ZX830. Each point represents one independent plant. For each plant, 500 pollen grains were examined. Bars indicate mean ± SD. Statistical significance was assessed using a two‐sided Welch's *t*‐test based on plant‐level abnormal pollen proportions. *n* = 3 independent plants per genotype (**p < 0.01). (P) Immunostaining of rye‐specific centromeric retrotransposon CRT1 (red) on meiotic chromosomes during Metaphase I, Anaphase I, Telophase II, and tetrad formation. Scale bars: 5 μm. (Q) Models of centromere evolution and R‐subgenome‐associated meiotic instability in hexaploid triticale. PC1 and PC2, the first and second principal components; SD, standard deviation; SNP, single‐nucleotide polymorphism.

To characterize population‐level genome divergence in triticale, we generated approximately 15 Tb of whole‐genome resequencing data from 99 triticale accessions and integrated these with resequencing datasets from tetraploid wheat and rye (Figure 2A, Figure S9, Table S9). Because triticale populations frequently contain chromosome substitutions and aneuploid lines [15], we first classified accessions based on read‐mapping profiles and cytological analyses. This revealed substantial karyotypic diversity, including canonical hexaploid lines, substitution lines, and aneuploids (Figures S10–S12). Principal component analysis (PCA) of ~61 million high‐quality SNPs showed that most canonical lines formed a tight cluster, whereas all substitution lines (except 4D → 4B), together with some canonical lines, formed a distinct, more dispersed cluster. This indicates that chromosomal substitutions contribute to marked genomic divergence, and the distribution of canonical lines within the dispersed cluster reflected geographic origin (Figure 2B). Integrating resequencing data from tetraploid wheat and rye, PCA separated triticale, tetraploid wheat, and rye into distinct groups, consistent with the hybrid origin of hexaploid triticale from wheat and rye ancestors and its composite genome structure (Figure 2C).

To determine whether genome‐wide population divergence in hexaploid triticale is accompanied by variation at functional centromeres, we performed pan‐centromere analyses using CENH3 ChIP‐seq datasets from 10 triticale accessions representing relatively diverse genetic backgrounds and geographic origins (Figure 2B, Tables S9 and S10). Relative to ZX830, many centromeres exhibited substantial structural variation, including narrowing, fragmentation, positional shifts, and complete loss of detectable CENH3 enrichment (Figure 2D, Figures S13 and S14). These alterations were strongly enriched in the R subgenome, whereas centromeres from the A and B subgenomes remained comparatively stable (Figure 2E,F). Interestingly, CEN4B lacked a detectable CENH3 peak in ~70% of accessions, consistent with its atypical repeat composition and extensive repeat turnover (Figure 2D). Collectively, these findings indicate that centromere variation represents an additional layer of genomic diversity in triticale and that the R subgenome is the principal axis of centromeric diversification during postpolyploid evolution.

## STRUCTURAL AND CENTROMERE‐ASSOCIATED DIVERGENCE IN THE R SUBGENOME IS ASSOCIATED WITH MEIOTIC DEFECTS AND STERILITY IN DIVERGENT TRITICALE HYBRIDS

Among all analyzed accessions, one line (PT) displayed the strongest centromeric divergence relative to ZX830, particularly within the R subgenomes (Figure 2F). PT also displayed a relatively large genetic distance from ZX830 in the PCA analysis (Figure 2B). We therefore generated a near‐complete chromosome‐scale assembly for PT using PacBio HiFi and Hi‐C sequencing (Figure 2G–I). The final assembly spanned 16.8 Gb, with 99.8% of sequences anchored into 21 pseudochromosomes and a scaffold N50 of 772.7 Mb (Tables S1–S5). Comparative analyses between ZX830 and PT revealed largely conserved syntenic relationships in the A and B subgenomes, whereas the R subgenome exhibited extensive structural divergence, with disrupted collinearity and pronounced rearrangements, particularly in centromeric and pericentromeric regions (Figure 2J–M, Figure S15, and Table S11). These observations are consistent with the elevated centromere variability detected in the pan‐centromere analysis.

To determine whether this divergence influences chromosome behavior, we generated PT **×** ZX830 F_1_ hybrids and examined male meiosis cytologically. In contrast to the normal meiotic progression observed in ZX830, F_1_ hybrids displayed severe abnormalities, including chromosome misalignment at metaphase I, lagging chromosomes during anaphase I, and micronuclei formation at the tetrad stage (Figure 2N). Consistently, pollen fertility was dramatically reduced in the hybrids relative to the parental lines (Figure 2O). Fluorescence in situ hybridization using CRT1 probe demonstrated that most lagging chromosomes and micronuclei originated from the R subgenome, suggesting that R‐subgenome divergence is associated with chromosome mis‐segregation during meiosis (Figure 2P). These results indicate that structural and centromeric divergence in the R subgenome constitutes an important genomic barrier contributing to reproductive isolation during triticale diversification.

Centromeres are often considered highly conserved functional domains despite rapid sequence evolution. Our analyses reveal that centromeres in triticale remain highly dynamic long after allopolyploid formation and identify the R subgenome as the primary source of structural and centromeric divergence during triticale evolution. The strong association between R‐subgenome divergence and meiotic instability further suggests that centromere remodeling contributes directly to postzygotic reproductive isolation in allopolyploid genomes (Figure 2Q).

This study has several limitations. Although our analyses establish an association between R‐subgenome divergence and centromeric variation with meiotic instability, the precise molecular mechanisms linking centromere remodeling to chromosome segregation defects remain unclear. In addition, the pan‐centromere analysis was conducted using a limited number of representative accessions, and broader sampling across geographically and genetically diverse triticale populations will be important to fully capture the global spectrum of centromere variation. Furthermore, the synthetic triticale analyzed in this study represents only an early postpolyploidization generation (S3), limiting our ability to fully resolve the temporal dynamics of centromere remodeling during long‐term polyploid evolution. Nevertheless, our work provides a genomic framework for understanding centromere evolution, genome stabilization, and reproductive isolation in allopolyploid species.

## AUTHOR CONTRIBUTIONS

**Yang Liu**: Conceptualization; investigation; validation; formal analysis; visualization; project administration; writing—original draft; writing—review and editing. **Wanna He**: Investigation; validation; formal analysis; visualization. **Chunhui Wang**: Investigation; validation; formal analysis; visualization. **Congyang Yi**: Investigation; validation; formal analysis; visualization. **Tianxin Guo**: Investigation; validation; formal analysis; visualization. **Congle Zhu**: Formal analysis. **Qian Liu**: Formal analysis. **Fangpu Han**: Supervision; funding acquisition; resources; project administration.

## CONFLICT OF INTEREST STATEMENT

The authors declare no conflicts of interest.

## ETHICS STATEMENT

No animals or humans were involved in this study.

## Supporting information


**Figure S1:** Near‐complete genome assembly of ZX830.
**Figure S2:** Subgenome composition and repetitive landscape of ZX830 and PT.
**Figure S3:** Centromere architecture and composition in ZX830.
**Figure S4:** Genome‐wide distribution and subgenome bias of LTR retrotransposon lineages in ZX830.
**Figure S5:** Centromeric landscape in ZX830.
**Figure S6:** Structural context of centromere size reduction on chromosomes 2R, 3R, and 4R in hexaploid triticale.
**Figure S7:** Structural context of centromere size reduction on chromosomes 5R, 6R, and 7R in hexaploid triticale.
**Figure S8:** Centromere repositioning and sequence organization at CEN1B in ZX830.
**Figure S9:** Grain morphology of 81 stable hexaploid triticale accessions.
**Figure S10:** Read‐depth profiles confirming canonical hexaploid karyotypes in triticale.
**Figure S11:** Read‐depth profiles identifying substitution lines and aneuploid accessions in triticale.
**Figure S12:** Cytological validation of karyotypic composition in representative triticale accessions.
**Figure S13:** CENH3 ChIP‐seq profiles of A‐ and B‐subgenome centromeres across ten hexaploid triticale accessions.
**Figure S14:** CENH3 ChIP‐seq profiles of R subgenome centromeres across ten hexaploid triticale accessions.
**Figure S15:** Structural comparison of centromeric and pericentromeric regions between PT and ZX830 across all chromosomes.

**Table S1:** Summary of HiFi data used in this research.
**Table S2:** Summary of Hi‐C data used in this research.
**Table S3:** The mapping rate of reads for ZX830 and PT genome.
**Table S4:** BUSCO assessment of genome assembly completeness for ZX830 and PT.
**Table S5:** Global statistics of ZX830 and PT genome assembly and annotation.
**Table S6:** Positions of functional CENH3‐binding regions in ZX830.
**Table S7:** Counts of different LTR retrotransposon families across chromosomes.
**Table S8:** Details of repeat family clusters identified in the CEN4B and CEN3B.
**Table S9:** Summary of 99 triticale germplasm resources.
**Table S10:** Genome assemblies and sequencing datasets used in this study.
**Table S11:** K‐mer‐based genome size estimation for ZX830 and PT using PacBio HiFi reads.

## Data Availability

The data that support the findings of this study are openly available in the National Genomics Data Center at https://bigd.big.ac.cn/, reference number PRJCA062163. All the sequencing data have been deposited in GSA/NGDC under BioProject accession number PRJCA062163 (PacBio HiFi and Illumina sequencing, https://ngdc.cncb.ac.cn/search/specific?=biosample&q=PRJCA062163). The genome assembly and annotation data have been archived at figshare (https://doi.org/10.6084/m9.figshare.32569086). The data and scripts used are saved in GitHub https://github.com/liuyang321/Centromeric-evolution-and-reproductive-isolation-in-hexaploid-triticale. Supplementary materials (methods, figures, tables, graphical abstract, slides, videos, Chinese translated version and update materials) may be found in the online DOI or iMeta Science http://www.imeta.science/.

## References

[1] Doyle, Jeff J. , Lex E. Flagel , Andrew H. Paterson , Ryan A. Rapp , Douglas E. Soltis , Pamela S. Soltis , and Jonathan F. Wendel . 2008. “Evolutionary Genetics of Genome Merger and Doubling in Plants.” Annual Review of Genetics 42: 443–461. 10.1146/annurev.genet.42.110807.091524 18983261

[2] Han, Xu , Xu , Jiahao Li , Guo Li , Zhibin Zhang , Taotao Lian , Bingqi Zhang , Ting Luo , et al. 2025. “Rapid Formation of Stable Autotetraploid Rice from Genome‐Doubled F_1_ Hybrids of *Japonica‐Indica* Subspecies.” Nature Plants 11: 743–760. 10.1038/s41477-025-01966-2 40164786 PMC12015120

[3] Steckenborn, Stefan , and André Marques . 2025. “Centromere Diversity and Its Evolutionary Impacts on Plant Karyotypes and Plant Reproduction.” New Phytologist 245: 1879–1886. 10.1111/nph.20376 39763092 PMC11798908

[4] Liu, Yang , Qian Liu , Congyang Yi , Chang Liu , Qinghua Shi , Mian Wang , and Fangpu Han . 2025. “Past Innovations and Future Possibilities in Plant Chromosome Engineering.” Plant Biotechnology Journal 23: 695–708. 10.1111/pbi.14530 39612312 PMC11869185

[5] Naish, Matthew , and Ian R. Henderson . 2024. “The Structure, Function, and Evolution of Plant Centromeres.” Genome Research 34: 161–178. 10.1101/gr.278409.123 38485193 PMC10984392

[6] Liu, Yang , Congyang Yi , Chaolan Fan , Qian Liu , Shulin Liu , Lisha Shen , Kaibiao Zhang , et al. 2023. “Pan‐Centromere Reveals Widespread Centromere Repositioning of Soybean Genomes.” Proceedings of the National Academy of Sciences of the United States of America 120: e2310177120. 10.1073/pnas.2310177120 37816061 PMC10589659

[7] Schubert, Ingo . 2018. “What Is Behind ‘Centromere Repositioning’?” Chromosoma 127: 229–234. 10.1007/s00412-018-0672-y 29705818

[8] Hu, Guanjing , Zhenyu Wang , Zunzhe Tian , Kai Wang , Gaoxiang Ji , Xingxing Wang , Xianliang Zhang , et al. 2025. “A Telomere‐to‐Telomere Genome Assembly of Cotton Provides Insights Into Centromere Evolution and Short‐Season Adaptation.” Nature Genetics 57: 1031–1043. 10.1038/s41588-025-02130-4 40097785

[9] Liu, Qian , Congyang Yi , Zeyan Zhang , Handong Su , Chang Liu , Yuhong Huang , Wei Li , et al. 2023. “Non‐B‐form DNA Tends to Form in Centromeric Regions and Has Undergone Changes in Polyploid Oat Subgenomes.” Proceedings of the National Academy of Sciences of the United States of America 120: e2211683120. 10.1073/pnas.2211683120 36574697 PMC9910436

[10] Chen, Chuanye , Siying Wu , Yishuang Sun , Jingwei Zhou , Yiqian Chen , Jing Zhang , James A. Birchler , Fangpu Han , Ning Yang , and Handong Su . 2024. “Three Near‐Complete Genome Assemblies Reveal Substantial Centromere Dynamics from Diploid to Tetraploid in Brachypodium Genus.” Genome Biology 25: 63. 10.1186/s13059-024-03206-w 38439049 PMC10910784

[11] Piro, Maria Chiara , Hans Peter Maurer , Hilde Muylle , Steven Maenhout , and Geert Haesaert . 2026. “A Genome‐Wide Association Study of Arabinoxylan Content in Flour of Triticale (*×Triticosecale* Wittmack).” Frontiers in Plant Science 17: 1809699. 10.3389/fpls.2026.1809699 42125608 PMC13158209

[12] De Zutter, Anneleen , Maria Chiara Piro , Steven Maenhout , Hans Peter Maurer , Johan De Boever , Hilde Muylle , Isabel Roldán‐Ruiz , and Geert Haesaert . 2024. “Genome‐Wide Association Study for In Vitro Digestibility and Related Traits in Triticale Forage.” BMC Plant Biology 24: 223. 10.1186/s12870-024-04927-7 38539072 PMC10976741

[13] Pattison, Angela L. , Marie Appelbee , and Richard M. Trethowan . 2014. “Characteristics of Modern Triticale Quality: Glutenin and Secalin Subunit Composition and Mixograph Properties.” Journal of Agricultural and Food Chemistry 62: 4924–4931. 10.1021/jf405138w 24792750

[14] Ukalska, Joanna , and Wanda Kociuba . 2013. “Phenotypical Diversity of Winter Triticale Genotypes Collected in the Polish Gene Bank Between 1982 and 2008 With Regard to Major Quantitative Traits.” Field Crops Research 149: 203–212. 10.1016/j.fcr.2013.05.010

[15] Cao, Dong , Dongxia Wang , Shiming Li , Yun Li , Ming Hao , and Baolong Liu . 2022. “Genotyping‐by‐Sequencing and Genome‐Wide Association Study Reveal Genetic Diversity and Loci Controlling Agronomic Traits in Triticale.” Theoretical and Applied Genetics 135: 1705–1715. 10.1007/s00122-022-04064-5 35244733

[16] Chen, Mengen , Junlong Li , Yue Zhang , Yanmeng Ding , Wenjie Qin , Mengyuan Wu , Na Li , et al. 2025. “Chromosome‐Level Genome Assembly of the Durum Wheat Cultivar Langdon.” Scientific Data 12: 1372. 10.1038/s41597-025-05724-z 40769987 PMC12328727

[17] Yi, Congyang , Qian Liu , Congle Zhu , Chang Liu , Chen Zhou , Wanna He , Chunhui Wang , Jing Yuan , Yang Liu \, and Fangpu Han . 2025. “High‐Resolution Genome Assembly Reveals Retrotransposon‐Mediated Centromere Dynamics in Rye.” Genome Biology 26: 308. 10.1186/s13059-025-03792-3 41013584 PMC12465393

