# Supplementary material for: Two near‐complete assemblies reveal R‐subgenome structural and centromeric divergence associated with reproductive isolation in hexaploid triticale
Source: Imeta. 2026 Jul 13;5(4):e70155. doi: 10.1002/imt2.70155 (PMC13519091; doi:10.1002/imt2.70155)
Supplement: Supplementary file 1 [file IMT2-5-e70155-s002.docx]

Supporting information to

**Two near-complete assemblies reveal R-subgenome structural and centromeric divergence associated with** **reproductive isolation in hexaploid triticale**

**Running title**: Centromere evolution and reproductive isolation in triticale

**Yang Liu^1^**^#^**, Wanna He^1, 2^**^#^**, Chunhui Wang^1^**^#^**, Congyang Yi^1^**^#^**, Tianxin Guo^1, 2^, Congle Zhu^1, 2^, Qian Liu^1^, Fangpu Han^1, 2*^**

**^1^**State Key Laboratory of Seed Innovation, **Institute of Genetics and Developmental Biology, Chinese Academy of Sciences, Beijing, 100101, China**

**^2^University of Chinese Academy of Sciences, Beijing, 100049, China**

^#^ **These authors contributed equally: Yang Liu, Wanna He, Chunhui Wang, Congyang Yi.**

**^*^**Correspondence**:** [fphan@genetics.ac.cn](mailto:fphan@genetics.ac.cn) **(Fangpu Han)**

**METHODS**

**Plant materials and growth conditions**

Triticale accessions used in this study were obtained from the U.S. National Plant Germplasm System (NPGS) and our institutional germplasm collection. These materials represent historically bred hexaploid triticale lines and the exact parental combinations used during original hybridization are not fully documented. Therefore, the AABB (tetraploid wheat) and RR (rye) genomic components in these accessions may derive from multiple widely used breeding lineages rather than a single defined cross. Triticale accessions were cultivated in a greenhouse at the Institute of Genetics and Developmental Biology, Chinese Academy of Sciences (Beijing, China; 40.22° N, 116.23° E). Seeds were sown in a substrate composed of commercial soil (Pindstrup, Denmark) mixed with vermiculite and grown at 25 °C under a controlled photoperiod of 16 h light and 8 h dark. Leaf tissues were harvested from 14-day-old seedlings, immediately flash-frozen in liquid nitrogen, and stored at −80 °C until further use.

**DNA library construction and sequencing**

For PacBio HiFi sequencing, SMRTbell libraries were constructed using the Pacific Biosciences SMRTbell Express Template Prep Kit 2.0 according to the manufacturer’s instructions. Size-selected DNA fragments with an average length of approximately 15 kb were used to generate SMRT Cell libraries, which were sequenced on the PacBio Sequel II platform with 30-hour movie collections.

For Hi-C library preparation, high-quality genomic DNA was isolated from young leaf tissue. Chromatin was cross-linked with formaldehyde, and in situ Hi-C libraries were prepared using a DNase I-based protocol. The resulting Hi-C libraries were sequenced on the Illumina NovaSeq platform to generate paired-end reads of 150 bp.

All library construction and sequencing were performed by Wuhan Benagen Technology Co., Ltd. (Wuhan, China).

**Genome assembly**

The PacBio HiFi data were *de novo* assembled using Hifiasm (v0.24.0-r702) [1] following recommended parameters for allopolyploid plant genomes. To improve assembly accuracy, we first removed redundant haplotypic/duplicated contigs using purge_dups (v1.2.5) [2] using parameters “-a 85” for coverage depth and sequence identity. We then screened for non-nuclear contaminants. Putative organellar sequences (chloroplast and mitochondrial) were identified by aligning contigs against published wheat organelle genomes using minimap2 (v2.26-r1175) with the asm5 preset (-x asm5) [3], and removed when alignments covered > 80% of the contig length. In addition, we constructed a decontamination step against non-plant reference databases, including curated archaeal (v5), bacterial (v1487), and viral (v1.1) genomes. Contigs exhibiting > 80% coverage to any non-plant entry were flagged and excluded using in-house Python scripts. This procedure yielded a filtered nuclear contig set for chromosomal scaffolding.

Chromosome-scale scaffolding was performed using Hi-C data. Hi-C reads were aligned to the filtered contig assembly using Chromap (v0.2.7-r494) [4] with default parameters. The resulting contact maps were used to order and orient contigs with YaHS (v1.2.2) [5] using the --no-contig-ec flag to disable automated contig correction. We then manually inspected and curated the scaffolds using Juicebox Assembly Tools (v1.11.08) [6], correcting misjoins and refining contig placements based on long-range interaction patterns. This step produced 21 chromosome-scale pseudomolecules. To reduce residual assembly gaps and local fragmentation, we applied iterative gap filling with RagTag2 (v2.1.0) [7]. Finally, we polished the assembly using NextPolish2 (v0.2.1) [8] with PacBio HiFi reads to correct residual indels and homopolymer errors and was subsequently used for all downstream analyses.

**Genome assembly evaluation and quality assessment**

Assembly quality was assessed using multiple complementary metrics to evaluate gene space completeness, repeat continuity, and large-scale structural integrity. Completeness of the assembled gene space was evaluated using Benchmarking Universal Single-Copy Orthologs (BUSCO v5.6.1) [9] with the embryophyta_odb10 dataset. BUSCO scores were summarized as the proportions of complete (single-copy and duplicated), fragmented, and missing orthologs, providing an estimate of the recovery of conserved plant genes. PacBio HiFi reads were aligned to the final assembly using minimap2 [3] with the -x map-hifi preset. For each dataset, we calculated the proportion of mapped reads and the breadth of genome coverage, defined as the fraction of assembled bases covered by at least one read. Coverage depth and uniformity were further examined to identify potential regions of sequence collapse or local overexpansion. Assembly quality within repetitive regions was evaluated using the LAI [10]. LAI scores were calculated with LTR_retriever (v1.1) [11] based on structurally intact LTR retrotransposons and were used to benchmark assembly continuity in highly repetitive genomic regions, including pericentromeric and centromeric domains. To assess scaffolding accuracy and large-scale structural correctness, Hi-C reads were processed with HiC-Pro (v3.1.0) [12] to generate chromatin contact matrices at 100-kb resolution. Contact heatmaps were examined to verify chromosome-scale interaction patterns and to detect potential misassemblies, such as interchromosomal chimeras, orientation errors, or local misjoins. Observations from Hi-C contact maps were used to guide the refinement of chromosome-scale pseudomolecules. Genome sizes of ZX830 and PT were estimated from PacBio HiFi reads using GenomeScope based on k-mer frequencies (k = 21, 31, and 51). Estimated genome size, sequencing coverage, model fit, error rate, and heterozygosity are summarized in Table S11.

**Repeat annotation**

Repetitive sequences in the genome assemblies were annotated using HiTE (v3.2) [13], an integrated pipeline designed for repeat identification in highly contiguous plant genomes. HiTE was executed with the parameters --plant 1 --recover 1 --annotate 1, using the Triticeae-specific CLARITE repeat library as a reference.

The annotation workflow combined multiple complementary strategies. *De novo* repeat discovery was performed using the HiTE-FMEA module, while terminal inverted repeats (TIRs), Helitrons, and non-LTR retrotransposons were further refined using the HiTE-TIR, HiTE-Helitron, and HiTE-NonLTR modules, respectively. Long terminal repeat (LTR) retrotransposons were identified based on structural features using LTR_FINDER, LTRharvest, and LTR_retriever.

All detected repetitive elements were consolidated into a non-redundant transposable element library and classified into major repeat superfamilies, including Gypsy, Copia, LINE, SINE, and DNA transposons, using RepeatClassifier and NeuralTE. The resulting repeat annotations were used to generate repeat-masked genome assemblies, which served as the basis for subsequent gene prediction and annotation analyses.

**Genome annotation**

Gene annotation was performed on the repeat-masked genome assemblies. Transcriptomic evidence for gene annotation was generated from RNA-seq data obtained from a pooled RNA sample comprising root, stem, leaf, and spike tissues. RNA-seq reads were aligned to the reference genome using HISAT2 (v2.2.1) [14], and transcript models were assembled with StringTie (v3.0.0) [15] using default parameters. For homology-based annotation, protein sequences from Triticeae species were retrieved from the NCBI database and clustered using CD-HIT [16] with a 95% sequence identity threshold to remove redundancy. The resulting non-redundant protein set was aligned to the genome using miniprot (v0.15-r270) [17], and splice-aware refinement of alignments was performed with gffread (v0.12.7) [18].

Transcript assemblies and protein alignments were integrated using EviAnno (v2.0.2) [19]. During evidence integration, duplicate transcript models were systematically identified and removed. Open reading frames (ORFs) predicted by TransDecoder (v5.7.1) were incorporated into the annotation pipeline, and splice junctions were evaluated using Markov chain-based scoring to validate transcript structures. Transcripts lacking protein homology were retained if intact ORFs were detected, whereas readthrough transcripts were flagged in the absence of premature stop codons. Protein-only loci without transcript support were excluded from downstream analyses. Following evidence integration, gene models were subjected to ORF repair and locus reassignment to improve structural accuracy. Pseudogenes were identified based on frameshift analyses. Final gene models were post-processed using AGAT (v1.4.1) to standardize gene identifiers, annotate untranslated regions (UTRs), and resolve any remaining redundant transcript models.

**Pan-centromere sampling and CENH3 ChIP-seq library preparation**

For pan-centromere analysis, ten hexaploid triticale accessions were selected to represent relatively diverse genetic backgrounds, geographic origins, and phenotypic characteristics within the global triticale population. These accessions originated from China, the United States, Ukraine, and Russia and occupied relatively distinct positions in the population structure analysis (Figure 2B and Table S9). To minimize technical variation among samples, all CENH3 ChIP-seq experiments were performed using the same experimental workflow, antibody conditions, sequencing platform, and downstream computational pipeline. Approximately 3–5 g of young leaf tissue was flash-frozen in liquid nitrogen and ground to a fine powder. Nuclei were extracted using lysis buffer 1 (1 mM EDTA, 50 mM HEPES pH 7.5, 150 mM NaCl, 1.0% Triton X-100, 10% glycerol, 5 mM β-mercaptoethanol, and 1× Complete Mini EDTA-free protease inhibitor cocktail). The isolated nuclei were pelleted and washed twice with lysis buffer 2 (1 mM EDTA, 50 mM HEPES pH 7.5, 150 mM NaCl, 1.0% Triton X-100, 10% glycerol, and 1× protease inhibitor cocktail), with centrifugation at 7000 × g for 3 min at 4 °C for each wash.

Nuclei were subsequently washed once in MNase digestion buffer (10% sucrose, 50 mM Tris-HCl pH 7.5, 1 mM CaCl_2_, 1 mM MgCl_2_, and 0.1 mM PMSF) and centrifuged at 7000 × g for 3 min at 4 °C. The chromatin pellet was resuspended in MNase buffer and digested with micrococcal nuclease (MNase; New England Biolabs, 0.5 U) at 37 °C for 24 min to generate chromatin fragments suitable for sequencing. The reaction was terminated by the addition of EDTA (final concentration 0.5 M), followed by centrifugation at 12,000 rpm for 3 min at 4 °C.

An aliquot of the supernatant was retained as the input control, while the remaining chromatin was incubated with anti-CENH3 antibody (1:400 dilution) at 4 °C overnight. Immunocomplexes were captured using 50 μL of protein A magnetic beads and sequentially washed with wash buffer (50 mM Tris-HCl pH 7.5, 10 mM EDTA, 0.2 mM PMSF, and 1 × protease inhibitor cocktail) containing increasing NaCl concentrations (50, 100, and 150 mM).

Bound chromatin was eluted in elution buffer (50 mM NaCl, 20 mM Tris-HCl, 5 mM EDTA, and 2% SDS) at 65 °C for 40 min. Purified nucleic acids were resuspended in 15 μL TE buffer (pH 8.0). ChIP and input DNA were used for library construction with the NadPrep DNA Library Preparation Kit for Illumina (Nanodigmbio, cat. no. 1002101) and NadPrep UDI Adapter Kit Set C1 (Nanodigmbio, cat. no. 1003221), following the manufacturer’s instructions. Libraries were sequenced on the Illumina NovaSeq platform to generate 150-bp paired-end reads.

**ChIP-seq raw data processing, peak calling, and centromere state classification**

Adapter sequences and low-quality bases were trimmed from raw reads using Trimmomatic (v0.36) [20]. After quality filtering, the cleaned reads were mapped to the corresponding reference genome using BWA-MEM [21] with default settings. The resulting alignments were further processed with SAMtools (v1.3.1) [22] to remove non-uniquely mapped reads and PCR duplicates. For downstream visualization, filtered BAM files were converted into normalized genome-wide coverage tracks (bigWig format) with a bin size of 5 bp using deepTools (v3.5.1) [23]. CENH3 ChIP-seq enrichment profiles were visualized using the Integrative Genomics Viewer (IGV) [24]. Functional centromere regions were identified from CENH3 ChIP-seq profiles using the matched input libraries as controls. For each accession, both ChIP and input reads were mapped to its corresponding genome assembly, and reads with mapping quality scores below 20 were discarded. Genome-wide ChIP and input signals were then converted into RPKM-normalized coverage profiles at 1-kb resolution using deepTools. To obtain robust enrichment patterns, the normalized signals were further summarized in 100-kb sliding windows with a 2-kb step size. For each window, the total ChIP signal and the ChIP-to-input enrichment ratio were calculated. Windows were considered candidate CENH3-enriched regions when they met two criteria simultaneously: ChIP signal above the genome-wide 99th percentile and ChIP/input enrichment above the 95th percentile. Neighboring candidate windows separated by ≤1 Mb were combined into continuous enriched regions, and only merged regions longer than 3 Mb were retained as putative functional centromeres. The final centromere boundaries were assigned according to the start and end positions of these merged CENH3-enriched regions.

Centromere structural states were classified by comparing each chromosome-level functional centromere with the corresponding canonical centromere in the ZX830 reference. Centromeres were classified as intact when the CENH3-enriched domain occurred at the syntenic centromere position and showed a comparable domain structure to the reference. Centromeres with conserved positions but markedly expanded or reduced CENH3-enriched domains were classified as widened or narrowed, respectively. Centromeres were classified as shifted when the major CENH3-enriched domain was displaced from the corresponding reference centromere region. Centromeres were classified as broken when two or more separated CENH3-enriched domains were detected within the corresponding centromeric region. Centromeres were classified as absent when no CENH3-enriched domain satisfying the peak-calling criteria was detected in the expected syntenic centromere region. Automated classifications were further inspected manually using ChIP and input coverage tracks to minimize misclassification caused by local mapping bias, low coverage, or assembly ambiguity.

**Synteny and collinearity**

Whole-genome alignments were generated using MUMmer (v4.0) [25] with the parameters --mum --mincluster 1000 --minmatch 100. The resulting pairwise alignments were subsequently analyzed with the SyRI pipeline (v1.5.3) [26] to systematically identify structural variants, including insertions, deletions, inversions, and translocations.

**Immunolocalization and FISH**

Immunolocalization and FISH were performed as described [27]. For immunostaining analyses, polyclonal anti-CENH3 antibodies were generated using antigenic peptides designed based on the CENH3 protein sequences from wheat and rye. For FISH, mitotic chromosome spreads were prepared from root apical meristems. The PCR-amplified DNAs were labeled with Alexa Fluor 488-dUTP (green) or Alexa Fluor 594-5-dUTP (red) as needed using the nick-translation method. Fluorescence signals were captured using a confocal microscope (Zeiss Cell Observer SD). Image processing and analysis were performed with ZEN 2009 Light Edition (Zeiss) and Adobe Photoshop CS6.

**SNP calling and population structure analysis**

Raw paired-end resequencing reads from 99 triticale accessions were quality-filtered using fastp. Clean reads were aligned to the ZX830 reference genome using BWA-MEM, sorted with SAMtools, and PCR duplicates were marked using Sambamba. Per-sample genomic VCFs were generated using GATK HaplotypeCaller in GVCF mode and jointly genotyped using CombineGVCFs and GenotypeGVCFs. Biallelic SNPs were retained using BCFtools and subjected to hard filtering using the criteria QUAL < 30, QD < 2.0, MQ < 40, FS > 60.0, SOR > 3.0, MQRankSum < −12.5, and ReadPosRankSum < −8.0. SNPs with minor allele frequency <0.05 or genotype calls in fewer than 90% of accessions were further removed using VCFtools, yielding 61,044,386 high-quality SNPs for downstream analyses.

For population structure analysis, biallelic SNPs were linkage-disequilibrium pruned in PLINK2 using --indep-pairwise 50 10 0.2, and principal component analysis was performed on the pruned SNP set using --pca 10. Pairwise genetic distances were calculated in PLINK using the --distance square option, and neighbor-joining trees were constructed from the resulting symmetric distance matrix using the nj() function in the R package ape. Chromosome-level read-depth profiles and cytological analyses were used to distinguish canonical AABBRR lines, chromosome-substitution lines, and aneuploid accessions during interpretation of the population structure results.

**Statistical analysis**

All statistical analyses were performed in R (v4.3.2). Differences in functional centromere size among Langdon, Weining, ZX830, and S3 were assessed using one-way analysis of variance (ANOVA) followed by post hoc pairwise comparisons. Differences in centromere divergence scores among the A-, B-, and R-subgenomes were likewise evaluated using one-way ANOVA followed by post hoc pairwise comparisons with Benjamini-Hochberg false discovery rate (FDR) correction for multiple testing. The proportion of abnormal pollen in PT × ZX830 F_1_ hybrids was compared using a two-sided Welch’s *t*-test based on plant-level abnormal pollen proportions. *n* = 3 independent plants per genotype. Boxplots were generated using the ggplot2 package, and statistical significance was denoted as *p* < 0.05 (*) and *p* < 0.01 (**).

**REFERENCES**

1. Cheng, Haoyu, Gregory T Concepcion, Xiaowen Feng, Haowen Zhang, Heng Li. 2021. “Haplotype-resolved de novo assembly using phased assembly graphs with hifiasm.” *Nature Methods* 18: 170-175. <https://doi.org/10.1038/s41592-020-01056-5>

2. Guan, Dengfeng, Shane A McCarthy, Jonathan Wood, Kerstin Howe, Yadong Wang, Richard Durbin. 2020. “Identifying and removing haplotypic duplication in primary genome assemblies.” *Bioinformatics* 36: 2896-2898. <https://doi.org/10.1093/bioinformatics/btaa025>

3. Li, Heng. 2018. “Minimap2: pairwise alignment for nucleotide sequences.” *Bioinformatics* 34: 3094-3100. <https://doi.org/10.1093/bioinformatics/bty191>

4. Zhang, Haowen, Li Song, Xiaotao Wang, Haoyu Cheng, Chenfei Wang, Clifford A Meyer, Tao Liu, et al. 2021. “Fast alignment and preprocessing of chromatin profiles with Chromap.” *Nature Communications* 12: 6566. <https://doi.org/10.1038/s41467-021-26865-w>

5. Zhou, Chenxi, Shane A McCarthy, Richard Durbin. 2023. “YaHS: yet another Hi-C scaffolding tool.” *Bioinformatics* 39(1): btac808. <https://doi.org/10.1093/bioinformatics/btac808>

6. Durand, Neva C, James T Robinson, Muhammad S Shamim, Ido Machol, Jill P Mesirov, Eric S Lander, Erez Lieberman Aiden. 2016. “Juicebox provides a visualization system for Hi-C contact maps with unlimited zoom.” *Cell Systems* 3: 99-101. <https://doi.org/10.1016/j.cels.2015.07.012>

7. Alonge, Michael, Ludivine Lebeigle, Melanie Kirsche, Katie Jenike, Shujun Ou, Sergey Aganezov, Xingang Wang, Zachary B Lippman, Michael C Schatz, Sebastian Soyk. 2022. “Automated assembly scaffolding using RagTag elevates a new tomato system for high-throughput genome editing.” *Genome Biology* 23: 258. <https://doi.org/10.1186/s13059-022-02823-7>

8. Hu, Jiang, Zhuo Wang, Fan Liang, Shan-Lin Liu, Kai Ye, De-Peng Wang. 2024. “NextPolish2: a repeat-aware polishing tool for genomes assembled using HiFi long reads.” *Genomics Proteomics Bioinformatics* 22(1): qzad009. <https://doi.org/10.1093/gpbjnl/qzad009>

9. Seppey, Mathieu, Mosè Manni, Evgeny M Zdobnov. 2019. “BUSCO: assessing genome assembly and annotation completeness.” *Methods in Molecular Biology* 1962: 227-245. <https://doi.org/10.1007/978-1-4939-9173-0_14>

10. Ou, Shujun, Jinfeng Chen, Ning Jiang. 2018. “Assessing genome assembly quality using the LTR Assembly Index (LAI).” *Nucleic Acids Research* 46: e126. <https://doi.org/10.1093/nar/gky730>

11. Ou, Shujun, Ning Jiang. 2018. “LTR_retriever: a highly accurate and sensitive program for identification of long terminal repeat retrotransposons.” *Plant Physiology* 176: 1410-1422. <https://doi.org/10.1104/pp.17.01310>

12. Servant, Nicolas, Nelle Varoquaux, Bryan R Lajoie, Eric Viara, Chong-Jian Chen, Jean-Philippe Vert, Edith Heard, Job Dekker, Emmanuel Barillot. 2015. “HiC-Pro: an optimized and flexible pipeline for Hi-C data processing.” *Genome Biology* 16: 259. <https://doi.org/10.1186/s13059-015-0831-x>

13. Hu, Kang, Peng Ni, Minghua Xu, You Zou, Jianye Chang, Xin Gao, Yaohang Li, Jue Ruan, Bin Hu, Jianxin Wang. 2024. “HiTE: a fast and accurate dynamic boundary adjustment approach for full-length transposable element detection and annotation.” *Nature Communications* 15: 5573. <https://doi.org/10.1038/s41467-024-49912-8>

14. Kim, Daehwan, Joseph M Paggi, Chanhee Park, Christopher Bennett, Steven L Salzberg. 2019. “Graph-based genome alignment and genotyping with HISAT2 and HISAT-genotype.” *Nature Biotechnology* 37: 907-915. <https://doi.org/10.1038/s41587-019-0201-4>

15. Pertea, Mihaela, Geo M. Pertea, Corina M. Antonescu, Tsung-Cheng Chang, Joshua T. Mendell, Steven L. Salzberg. 2015. “StringTie enables improved reconstruction of a transcriptome from RNA-seq reads.” *Nature Biotechnology* 33: 290-295. <https://doi.org/10.1038/nbt.3122>

16. Li, Weizhong, Adam Godzik. 2006. “Cd-hit: a fast program for clustering and comparing large sets of protein or nucleotide sequences.” *Bioinformatics* 22: 1658-1659. <https://doi.org/10.1093/bioinformatics/btl158>

17. Li, Heng. 2023. “Protein-to-genome alignment with miniprot.” *Bioinformatics* 39(1):btad014. <https://doi.org/10.1093/bioinformatics/btad014>

18. Pertea, Geo, Mihaela Pertea. 2020. “GFF Utilities: GffRead and GffCompare.” *F1000Research* 9: 304. <https://doi.org/10.12688/f1000research.23297.2>

19. Zimin, Aleksey V, Daniela Puiu, Mihaela Pertea, James A Yorke, Steven L Salzberg. 2025. “Efficient evidence-based genome annotation with EviAnn.” *bioRxiv* <https://doi.org/10.1101/2025.05.07.652745>

20. Bolger, Anthony M, Marc Lohse, Bjoern Usadel. 2014. “Trimmomatic: a flexible trimmer for Illumina sequence data.” *Bioinformatics* 30: 2114-2120. <https://doi.org/10.1093/bioinformatics/btu170>

21. Li, Heng, Richard Durbin. 2009. “Fast and accurate short read alignment with Burrows-Wheeler transform.” *Bioinformatics* 25: 1754-1760. <https://doi.org/10.1093/bioinformatics/btp324>

22. Li, Heng, Bob Handsaker, Alec Wysoker, Tim Fennell, Jue Ruan, Nils Homer, Gabor Marth, Goncalo Abecasis, Richard Durbin. 2009. “The sequence alignment/map format and SAMtools.” *Bioinformatics* 25: 2078-2079. <https://doi.org/10.1093/bioinformatics/btp352>

23. Ramírez, Fidel, Devon P Ryan, Björn Grüning, Vivek Bhardwaj, Fabian Kilpert, Andreas S Richter, Steffen Heyne, Friederike Dündar, Thomas Manke. 2016. “deepTools2: a next generation web server for deep-sequencing data analysis.” *Nucleic Acids Research* 44: W160-165. <https://doi.org/10.1093/nar/gkw257>

24. Thorvaldsdóttir, Helga, James T Robinson, Jill P Mesirov. 2013. “Integrative Genomics Viewer (IGV): high-performance genomics data visualization and exploration.” *Briefings in Bioinformatics* 14: 178-192. <https://doi.org/10.1093/bib/bbs017>

25. Marçais, Guillaume, Arthur L Delcher, Adam M Phillippy, Rachel Coston, Steven L Salzberg, Aleksey Zimin. 2018. “MUMmer4: a fast and versatile genome alignment system.” *PLoS Computational Biology* 14: e1005944. <https://doi.org/10.1371/journal.pcbi.1005944>

26. Goel, Manish, Hequan Sun, en-Biao Jiao, Korbinian Schneeberger. 2019. “SyRI: finding genomic rearrangements and local sequence differences from whole-genome assemblies.” *Genome Biology* 20: 277. <https://doi.org/10.1186/s13059-019-1911-0>

27. Liu, Yang, Handong Su, Yalin Liu, Jing Zhang, Qianhua Dong, James A Birchler, Fangpu Han. 2017. “Cohesion and centromere activity are required for phosphorylation of histone H3 in maize.” *Plant Journal* 92: 1121-1131. <https://doi.org/10.1111/tpj.13748>

 **Figure S1 Near-complete genome assembly of ZX830.** (A) Karyotype of the hexaploid triticale line ZX830 showing 21 pairs of chromosomes. The left panel shows genomic *in situ* hybridization (GISH), with the R subgenome labeled in red, the A subgenome in green, and the B subgenome in light blue. The right panel shows fluorescence *in situ* hybridization (FISH) images from the same metaphase spread, using probes pSc119.2 (green) and pTa535 (red) to distinguish individual chromosomes. Scale bars: 10 μm. (B) Chromatin interaction map generated by Hi-C for the ZX830 genome. (C) Chromosome-scale synteny analyses between hexaploid triticale ZX830 and its parental species. The A and B subgenomes of ZX830 show high collinearity with those of tetraploid durum wheat (*Triticum turgidum* cv. Langdon, AABB), whereas the R subgenome exhibits strong collinearity with cultivated rye (*Secale cereale* cv. Weining, RR).

 **Figure S2** **Subgenome composition and repetitive landscape of ZX830 and PT.** (A) Proportion of repetitive sequences in the A, B, and R subgenomes of hexaploid triticale lines ZX830 and PT. (B) Composition of major classes of repetitive elements in the triticale genome, including LTR retrotransposons, DNA transposons, and other repeat categories, shown as percentages of the total genome for ZX830 and PT. (C) Subgenome-level comparison of genome size, gene number, and gene density in ZX830. Bars indicate genome size (blue) and gene number (orange), while the line represents gene density. (D) Subgenome-level comparison of genome size, gene number, and gene density in PT, shown using the same representation as in (C). (E) Total length of intronic regions in the A, B, and R subgenomes of ZX830 and PT. (F) Total length of intergenic regions in the A, B, and R subgenomes of ZX830 and PT.

 **Figure S3** **Centromere architecture and composition in ZX830.** (A) Multiple sequence alignment of CENH3 homologs from major plant species. The alignment includes alpha- and beta-type CENH3 variants from rye (*Secale cereale*), wheat (*Triticum turgidum*), barley (*Hordeum vulgare*), as well as homologs from maize (*Zea mays*), rice (*Oryza sativa*), Arabidopsis (*A. thaliana and A. arenosa*), soybean (*Glycine max*), Medicago (*M. truncatula*), *Amphicarpaea edgeworthii*, and oat (*Avena sativa*). The highlighted peptide region corresponds to the epitope used for generating the anti-CENH3 polyclonal antibody. Key structural annotations, including the variable N-terminal tail and the conserved CENP-A targeting domain (CATD), are indicated. (B) CENH3 ChIP-seq signals in triticale generated using wheat-specific (AABB tracks) and rye-specific (RR tracks) antibodies, as well as a mixed-antibody ChIP-seq dataset (Merge). CRT refers to a class of centromere-enriched retrotransposons identified in triticale. All reads were aligned to the ZX830 reference genome. Genome-wide enrichment profiles are shown in the upper panel. The lower panel displays a zoomed-in view of a representative region on chromosome 5B (chr5B), illustrating consistent CENH3 enrichment patterns across antibody types. (C) Genome-wide profiles of CENH3 ChIP-seq signal intensity across all 21 chromosomes of hexaploid triticale ZX830, generated using a combination of wheat- and rye-specific CENH3 antibodies.

 **Figure S4 Genome-wide distribution and subgenome bias of LTR retrotransposon lineages in ZX830**. (A) Stacked bars show the proportion of major sequence categories, including LTR retrotransposons, DNA transposons, simple repeats, and other sequences, for each CENH3-defined centromeric region. (B) Genome-wide distribution of CENH3 ChIP-seq signal, gene density, and major LTR retrotransposon lineages across the 21 chromosomes of hexaploid triticale ZX830. Tracks from top to bottom show CENH3 ChIP-seq signal intensity, gene density, and the distribution of centromere-associated CRM elements and representative LTR retrotransposon families, including Angela, Retand, SIRE, Tekay, Athila, and Ale. (C) Subgenome-specific abundance of major LTR retrotransposon lineages in hexaploid triticale. Bar plots show the copy number of each retrotransposon family in the A (blue), B (orange), and R (gray) subgenomes.

**Figure S5 Centromeric landscape in ZX830.** (A) Dot-plot comparisons of centromeric regions between the R subgenome and the A/B subgenomes. (B) Genomic distribution of centromere-associated retrotransposon lineages across all chromosomes. Tracks show CENH3 ChIP-seq signal, followed by the distribution of CRT1 and CRT2 elements.

**Figure S6 Structural context of centromere size reduction on chromosomes 2R, 3R, and 4R in hexaploid triticale**. Synteny analyses between the R subgenome of hexaploid triticale ZX830 and its rye progenitor Weining from Chr2R to Chr4R. For each panel, tracks from top to bottom show CENH3 ChIP-seq signal in third-generation synthesized triticale (S3), CENH3 ChIP-seq signal in ZX830, the distribution of CRT1 elements in ZX830, pairwise syntenic relationships between ZX830 and Weining, the distribution of CRT1 elements in Weining, and CENH3 ChIP-seq signal in Weining. Colored ribbons indicate different classes of structural relationships, including synteny, inversion, translocation, and duplication, as indicated in the legend. Analyses span the core centromere and approximately 100 Mb of flanking pericentromeric regions for each chromosome.

**Figure S7 Structural context of centromere size reduction on chromosomes 5R, 6R, and 7R in hexaploid triticale**. Synteny analyses between the R subgenome of hexaploid triticale ZX830 and its rye progenitor Weining from Chr5R to Chr7R. For each panel, tracks from top to bottom show CENH3 ChIP-seq signal in S3, CENH3 ChIP-seq signal in ZX830, the distribution of CRT1 elements in ZX830, pairwise syntenic relationships between ZX830 and Weining, the distribution of CRT1 elements in Weining, and CENH3 ChIP-seq signal in Weining. Colored ribbons indicate different classes of structural relationships, including synteny, inversion, translocation, and duplication, as indicated in the legend. Analyses span the core centromere and approximately 100 Mb of flanking pericentromeric regions for each chromosome.

**Figure S8 Centromere repositioning and sequence organization at CEN1B in ZX830**. (A) Detailed sequence composition of the CEN1B region in ZX830, showing CENH3 ChIP-seq signal, trinucleotide repeat distribution, LTR retrotransposons, and DNA transposons across the centromeric interval. (B) Comparison of trinucleotide repeat copy numbers within the left and right flanking blocks of the CEN1B region between Langdon and ZX830. Values represent direct repeat copy number counts identified from genome assemblies. (C) Synteny analysis between ZX830 and its tetraploid wheat progenitor Langdon across the CEN1B region and flanking sequences. Tracks show CENH3 ChIP-seq signals, simple repeats, centromere-associated LTR retrotransposons, and pairwise syntenic relationships. Gray, yellow, green, and blue ribbons represent syntenic blocks, inversions, translocations, and duplications, respectively.

**Figure S9 Grain morphology of 81 stable hexaploid triticale accessions**. Representative grain images of 81 stable hexaploid triticale accessions originating from different geographic regions. Accessions are grouped according to their country of origin. Arrows indicate chromosome substitution lines, including 4D/4B and 6D/6A substitutions, whereas the remaining accessions represent canonical hexaploid triticale lines (AABBRR) and 2D/2R substitutions. Scale bars: 1 cm.

**Figure S10 Read-depth profiles confirming canonical hexaploid karyotypes in triticale**. Genome-wide read-depth profiles of 63 canonical hexaploid triticale accessions (AABBRR) obtained by mapping resequencing reads to a combined reference genome (AABBDDRR). Columns correspond to individual chromosomes of the A, B, D, and R subgenomes, as indicated at the top, while rows represent individual accessions. Normalized read-depth signals show uniform coverage across all A, B, and R chromosomes, consistent with the expected hexaploid karyotype and absence of large-scale chromosomal gains or losses. Accession IDs are indicated on the left, with corresponding material names listed in Table S9.

**Figure S11 Read-depth profiles identifying substitution lines and aneuploid accessions in triticale**. (A) Genome-wide read-depth profiles of triticale substitution lines, obtained by mapping resequencing reads to the combined AABBDDRR reference genome. Rows represent individual accessions and columns correspond to chromosomes of the A, B, D, and R subgenomes, as indicated at the top. Colored tracks denote different types of chromosomal substitutions: 2D → 2R substitution lines (14 accessions), 6D → 6A substitution lines (3 accessions), and a 4D → 4B substitution line (1 accession). Accession 3 represents an octoploid triticale (AABBDDRR), showing elevated and uniform read depth across all A, B, D, and R chromosomes. (B) Genome-wide read-depth profiles of aneuploid triticale accessions. These accessions exhibit approximately half of the expected read depth across specific chromosomes or chromosome segments, consistent with chromosomal loss relative to the canonical hexaploid karyotype. Accession IDs are indicated on the left, with corresponding material names listed in Table S9.

**Figure S12 Cytological validation of karyotypic composition in representative triticale accessions.** Cytological characterization of representative triticale accessions using genomic in situ hybridization (GISH) and fluorescence *in situ* hybridization (FISH), combined with genome-wide read-depth profiles. (A) Canonical hexaploid triticale. (B) Substitution lines. (C) Aneuploid accessions. Each panel shows GISH images (left) with the R subgenome labeled in red, A subgenome in green, and B subgenome in light blue, and FISH images (right) of the same metaphase spreads using repetitive probes pSc119.2 (green) and pTa535 (red) to distinguish individual chromosomes. Genome-wide read-depth profiles for the corresponding accessions are displayed to the right, with coverage plotted across chromosomes of the A, B, D, and R subgenomes (indicated at top). Scale bars: 10 μm.

**Figure S13** **CENH3 ChIP-seq profiles of A- and B-subgenome centromeres across ten hexaploid triticale accessions.** Colors indicate centromere states: intact (green), widened (blue), shifted (red), narrowed (purple), broken (orange), fragmented (gray), and absent (white). Accessions are labeled S1–S9, where S1 corresponds to PT, S2 to PI628657, S3 to L20191165, S4 to L20210904, S5 to WH10-4, S6 to Asperis, S7 to NTH1888, S8 to Jin, and S9 to Y70; accession details are provided in Table S9.

**Figure S14 CENH3 ChIP-seq profiles of R subgenome centromeres across ten hexaploid triticale accessions.** Colors indicate centromere states: intact (green), widened (blue), shifted (red), narrowed (purple), broken (orange), fragmented (gray), and absent (white). Accessions are labeled S1–S9, where S1 corresponds to PT, S2 to PI628657, S3 to L20191165, S4 to L20210904, S5 to WH10-4, S6 to Asperis, S7 to NTH1888, S8 to Jin, and S9 to Y70; accession details are provided in Table S9.

**Figure S15 Structural comparison of centromeric and pericentromeric regions between PT and ZX830 across all chromosomes**. Pairwise comparisons of centromeric and pericentromeric regions between ZX830 and PT for chromosomes not shown in Figure 2K–M. For each chromosome, a 100-Mb region centered on the functional centromere was extracted for comparative analysis. Conserved syntenic blocks and structural rearrangements were identified based on genome alignments.
